# A Meta-Analysis on the Relationship between Self-Reported Presence and Anxiety in Virtual Reality Exposure Therapy for Anxiety Disorders

**DOI:** 10.1371/journal.pone.0096144

**Published:** 2014-05-06

**Authors:** Yun Ling, Harold T. Nefs, Nexhmedin Morina, Ingrid Heynderickx, Willem-Paul Brinkman

**Affiliations:** 1 Interactive Intelligence Group, Delft University of Technology, Delft, the Netherlands; 2 Department of Clinical Psychology, University of Amsterdam, Amsterdam, the Netherlands; 3 Human Technology Interaction Group, Eindhoven University of Technology, Eindhoven, the Netherlands; 4 Visual Experiences Group, Philips Research Laboratories, Eindhoven, the Netherlands; ICREA-University of Barcelona, Spain

## Abstract

In virtual reality exposure therapy (VRET) for anxiety disorders, sense of presence in the virtual environment is considered the principal mechanism that enables anxiety to be felt. Existing studies on the relation between sense of presence and level of anxiety, however, have yielded mixed results on the correlation between the two. In this meta-analysis, we reviewed publications on VRET for anxiety that included self-reported presence and anxiety. The comprehensive search of the literature identified 33 publications with a total of 1196 participants. The correlation between self-reported sense of presence and anxiety was extracted and meta-analyzed. Potential moderators such as technology characteristics, sample characteristics including age, gender and clinical status, disorder characteristics and study design characteristics such as measurements were also examined. The random effects analysis showed a medium effect size for the correlation between sense of presence and anxiety (*r* = .28; 95% CI: 0.18–0.38). Moderation analyses revealed that the effect size of the correlation differed across different anxiety disorders, with a large effect size for fear of animals (*r* = .50; 95% CI: 0.30–0.66) and a no to small effect size for social anxiety disorder (*r* = .001; 95% CI: −0.19–0.19). Further, the correlation between anxiety and presence was stronger in studies with participants who met criteria for an anxiety disorder than in studies with a non-clinical population. Trackers with six degrees of freedom and displays with a larger field of view resulted in higher effect sizes, compared to trackers with three degrees of freedom and displays with a smaller field of view. In addition, no difference in effect size was found for the type of presence measurement and the type of anxiety measurement. This meta-analysis confirms the positive relation between sense of presence and anxiety and demonstrates that this relation can be affected by various moderating factors.

## Introduction

Anxiety disorders have an estimated one-year prevalence rate of about 18% among Americans [1]. In the fourth and fifth edition of the Diagnostic and Statistical Manual of Mental Disorders (DSM-IV, DSM-IV-TR and DSM-V) [2]–[4], anxiety disorders are characterized as excessive fear and anxiety with related behavioral disturbances. Anxiety disorders include separation anxiety disorder, specific phobias, social anxiety disorder, panic disorder, agoraphobia, generalized anxiety disorder, obsessive-compulsive disorder (OCD), posttraumatic stress disorder (PTSD), substance/medication induced anxiety disorder, anxiety disorder due to another medical condition and other specified anxiety disorders. A common treatment for anxiety disorders is cognitive behavioral therapy, in which patients are exposed to anxiety-provoking situations, generally in real life or through imaginal exposure where patients are asked to imagine a situation they are afraid of. In the last two decades, exposure treatment has also been offered through virtual reality, referred to as virtual reality exposure therapy (VRET) [5].

Similar to exposure in vivo, patients undergoing VRET are subjected to anxiety-provoking stimuli in a gradual order, from the least anxiety provoking stimulus to the most anxiety provoking one [6]. Hence, particularly because VRET offers more control on the anxiety level of the stimulus, it is considered as a good, and very practical alternative to traditional exposure in vivo. Research has demonstrated that VRET is an effective intervention for a variety of anxiety disorders and is similarly effective as exposure in vivo, the latter being the golden standard for treatment of anxiety disorders [7]–[10].

In exposure therapy, emotional processing had been proposed as a mechanism of change by Foa and Kozak [11]. With regard to anxiety disorders, for emotional processing to take place, a fear structure needs to be activated. The activation of fear enables new and corrective information to be incorporated in the memory structure, leading to a change in fear response [11]. In VRET, the sense of presence has been considered the principal mechanism that leads to the experience of anxiety [12]. Therefore, understanding the relationship between presence and anxiety seems essential for VRET. Knowledge about this relation might help further improve the efficacy of VRET.

The concept of presence in virtual reality covers three aspects: spatial presence, social presence, and co-presence [13], [14]. Spatial presence refers to the sense of being physically located in the virtual environment rather than in the environment in which people are physically located [13]–[15]. Social presence refers to the feeling of being together and of social interaction with other beings, i.e., a synthetic or a remotely located communication partner [13]–[15]. Co-presence is defined as the feeling of being together with others in a computer-generated world at the same time even though people are in separate places. Co-presence is considered as the intersection of spatial presence and social presence [14]. In this meta-analysis, we included all these three types of presence, i.e., spatial presence, social presence and co-presence, in our literature search. Presence experienced in augmented reality (AR) is also included in this study.

Presence is usually measured using self-reported measurements. Typical presence questionnaires are Igroup Presence Questionnaire (IPQ) [16], Slater-Usoh-Steed Questionnaire (SUS) [17], [18], Presence Questionnaire (PQ) [19], [20] and Independent Television Commission (ITC) - Sense of Presence Inventory (ITC-SOPI) [21]. But these questionnaires only include items measuring spatial presence. In addition, presence is also measured by objective corroborative measurements including physiological measurements and behavioral observations [22]. Similarly, anxiety can be measured by self-reported measurements, physiological measurements, and behavioral observations [12]. In this study, we only focus on self-reported measurements for both presence and anxiety.

To our knowledge, there are no published quantitative syntheses on the relationship between presence and anxiety. Existing published studies show mixed results. While some studies have found significant positive correlations between self-reported presence and anxiety in VRET [23], [24], some have not [25], [26]. Some studies even found negative correlations between presence and anxiety [27], [28]. The principal consequence of presence is that a user can experience the same emotions and reactions within a virtual environment as would be expected in a similar real-world situation. However, efforts to increase the sense of presence have not always led to higher levels of anxiety [25]. Therefore, to what extent a higher level of self-reported presence can generate more self-reported anxiety is not clear so far. Because of this lack of clarity we conducted a meta-analytic review of publications on the relation between sense of presence and anxiety in VRET. Meta-analyses provide estimates of a population effect size across independent studies. Hence, it could facilitate a better understanding of the relationship between self-reported presence and anxiety in VRET. Factors such as participants' characteristics including age, gender and clinical status, technology characteristics, disorder characteristics and study design characteristics including measurements, sample size and publication year are different among studies. The association between sense of presence and anxiety might also be moderated by these factors.

So, in summary, this study employs a meta-analytic approach to examine the overall effect size of the relationship between presence and anxiety across studies. Factors that potentially might affect this relationship are included as moderators, such as participants' characteristics, i.e., age, gender and clinical status, technology characteristics, disorder characteristics and study design characteristics i.e., measurements, sample size and publication year.

## Methods

### 2.1 Study selection

We selected studies related to VRET for anxiety disorders as defined in DSM-IV-TR or earlier version of the DSM [3], [4]. The search was conducted for studies published all years up till March 19, 2013. We searched the following databases: PsycINFO, PubMed, Web of Knowledge, and Scopus. The search terms used for presence, anxiety and virtual reality, respectively, are presented in Table 1. Names of typical presence questionnaires, such as Independent Television Commission (ITC) - Sense of Presence Inventory (ITC-SOPI) [21], Igroup Presence Questionnaire (IPQ) [16], Slater-Usoh-Steed Questionnaire (SUS) [17], [18] and Presence Questionnaire (PQ) [19], [20], were additionally used as search terms in Scopus. The references from recent meta-analyses, and systematic reviews on virtual reality exposure therapy [7]–[10], [29]–[36] were further screened for potentially relevant publications. Finally, papers in the International Conference Series on Disability, Virtual Reality and Associated Technologies (ECDVRAT) were also screened for potentially relevant publications.

**Table 1:** Items included in the comprehensive database search.

Presence	Anxiety	Virtual reality
presence	anxiety	virtual reality
telepresence	fear	virtual environment
co-presence	disorder	computer world
copresence	disorders	computer simulated environment
realism	phobi*	artificial reality
immersi*	OCD	augmented reality
	PTSD	mediated reality
	*phobia	virtual simulator

The asterisk (*) represents a wildcard, i.e., any group of characters, including non-letter character.

The inclusion criteria were a combination of: (1) studies with human participants in virtual or augmented reality environments for treatment of anxiety disorders, (2) studies regarding subjective self-reported presence and anxiety, and (3) studies published in peer-reviewed journals or conference proceedings. No language restrictions were used.

The exclusion criteria were any of the following: (1) studies that used virtual or augmented reality environments not a priori created or selected to elicit anxiety related to the target disorder (e.g., Riva et al. [37]), (2) studies addressing anxiety distraction or relaxation, (3) case studies with less than 3 participants (note that in practice the smallest sample size included was N = 6), (4) studies using non-natural stressors as they have not been conducted in the context of VRET, e.g., specific color caused by fear conditioning using electric shock by Ewald et al. [38], and (5) studies having not enough data to calculate the correlation even after emailing the author for additional data.

First, titles and abstracts of potentially relevant publications were read to eliminate irrelevant studies. After reading the full article, studies that were rated by the first author of this paper as not clearly meeting the inclusion criteria were discussed with the other co-authors. We contacted the authors of 50 potentially relevant articles that included self-reported presence and anxiety measurement for information not reported in the publication (i.e., information about display technology and tracker, information about the participants, or values for the correlations between self-reported presence and anxiety). We further asked these authors if they could refer us to unpublished work potentially relevant for this meta-analysis.

### 2.2. Procedure

Data on the following variables were collected: the correlation coefficient between presence and anxiety, self-reported presence and anxiety measurements, anxiety disorder type, sample size, clinical status (i.e., meeting criteria for an anxiety disorder or not), age, gender, technology-related characteristics, and publication year. As two studies [39], [40] only recruited male participants, we used the proportion of male participants as a continuous variable to evaluate the effect of gender on the correlation between presence and anxiety. Specific phobias including fear of spider, cockroach, dog, snake and wasp were grouped together and coded as fear of animals. The technology related characteristics included display device type, presence of stereoscopy, display spatial resolution, the diagonal field of view (FOV), the audio type and device, and the tracker system. Two widely used virtual reality displays are head-mounted display (HMD) that has a small display in front of one eye or both eyes, and cave automatic virtual environment (CAVE) where projectors are directed to three to six of the walls of a room-sized cube. Most of the display devices can render stereoscopy which permits the perception of objects floating in front of or behind the screen plane. Tracker systems are used to capture translation coordinates (*x*,*y*,*z*) and yaw, pitch, roll rotation coordinates and can show the virtual environment to the viewer while tracking the viewer's body movement. Trackers systems that have freedom of motion in all six of these directions are called 6 DOF tracking devices. Trackers that can only track three dimensions, e.g., translation along two axes and rotation in only one direction, are called 3 DOF tracking devices. The data update frequency (Hz) of the tracking system is also essential for real-time tracking. Note that for CAVE systems with four sides we assigned a value of 270° for the FOV.

As mentioned before, the widely used presence questionnaires basically measure spatial presence. Although the literature search included all three types of presence, the publications that met the inclusion criteria, and therefore were included in the meta-analysis, only reported spatial presence. Presence questionnaires included in the meta-analysis were the ITC-Sense of Presence Inventory [21], the Igroup Presence Questionnaire [16], the Slater-Usoh-Steed Questionnaire [17], [18], the Presence Questionnaire [19], [20], the one-item presence question [23], [41] and others [42], [43]. Anxiety measurements included in the meta-analysis were the state-anxiety scale from the Strait-Trait Anxiety Inventory (STAI-S) [44], the Subjective Units of Discomfort scale (SUD) [45] and the one-item question [23], [26], [46].

Correlations between self-reported presence and anxiety were collected and used as effect size in the meta-analysis. For studies with multiple sessions of treatment in virtual reality, only the data of the first virtual reality exposure session were used. So, even for studies with a repeated design, only the correlation of a person's first exposure session was considered, making it possible to treat these data as between-group data. For studies with multiple measurements of presence (e.g., comparing the one-item question with other questionnaires), only the correlation based on the widely used presence questionnaires (i.e., IPQ, ITC-SOPI, PQ, SUS) was included in the meta-analysis. As earlier studies [27], [47] found no order effect on presence during one session, we assumed that presence was stable during participants' exposure to the virtual environment. Therefore, if presence was measured multiple times in a session, the correlation with the averaged presence score was used.

Due to habituation to the virtual stimuli in the virtual environment, participants' anxiety would decrease over time [48], [49]. Therefore, if anxiety was measured multiple times in a session, the correlation with the highest anxiety score was selected. When multiple anxiety measurements (SUD and STAI-S) were used, correlations with the highest anxiety score (SUD) in a session was considered first. If the highest SUD score was not available, the correlation based on STAI-S was included. If the correlation between the highest anxiety score in a session and presence was not available, the correlation between the averaged anxiety score and presence was included.

### 2.3 Effect size calculation

The correlation coefficient itself served as the effect size index. The effect size can be categorized as small (0.1), medium (0.3) and large (0.5) as suggested by Cohen [50]. For the meta-analyses, we first converted the correlations into Fisher's *z* scores and performed syntheses on these Fisher's *z* scores. All analyses were performed using the transformed values. The summary effect and its confidence interval were then converted back to correlations for presentation of the results. The summary effect size was calculated using the random effects model due to the heterogeneity of the studies.

We first computed the overall effect size in which the data from all the studies were taken together. We then also conducted additional analyses: i.e., subgroup analyses when the moderators were categorical data and regression analyses when the moderators were continuous. For the subgroup analyses, effects had to be demonstrated by at least two different research teams in each subgroup. We pooled within-group estimates of tau-squared, assuming a common among-study variance component across the subgroups [51]. As Viechtbauer [52] recommends using a restricted maximum likelihood variance estimator since it strikes a good balance between unbiasedness and efficiency, meta-regressions were performed using a random-effects model with a restricted maximum likelihood (REML) random effects regression model thereby using effect size as the dependent variable. Multi-regression analyses were also performed when including more than one moderator for either categorical or continuous moderators.

All analyses were completed with the Comprehensive Meta-analysis Statistical package Version 3. Note that in the random-effects model for calculating the overall effect size and the subgroup analyses, this software only provides method of moments (or the DerSimonian and Laird [53]) to estimate the between-studies variance [51].

### 2.4 publication bias

When performing a meta-analysis, there are concerns that studies with smaller effect sizes are missing (because they are less likely to be reported). Accordingly, an inclusion of unpublished studies in the meta-analysis might have an impact on the average *p*-value of all studies combined. A conservative method of addressing this problem is to assume that the effect size of all current or future unpublished studies is equal to zero and to compute the number of such studies it would require to reduce the overall effect size to a non-significant level (α = .05, two-tailed). To test whether the overall effect that we found was robust, the classic *Fail-safe N* value as proposed by Rosenthal [54] was calculated. Rosenthal [54] suggested that findings could be considered as robust if the required number of studies to reduce the overall effect size to a non-significant level exceeded 5 × number of included studies (effect sizes)+10. Publication bias was also assessed with a funnel plot visually, and qualified with Egger's weighted regression method [55].

## Results

**Figure 1:**
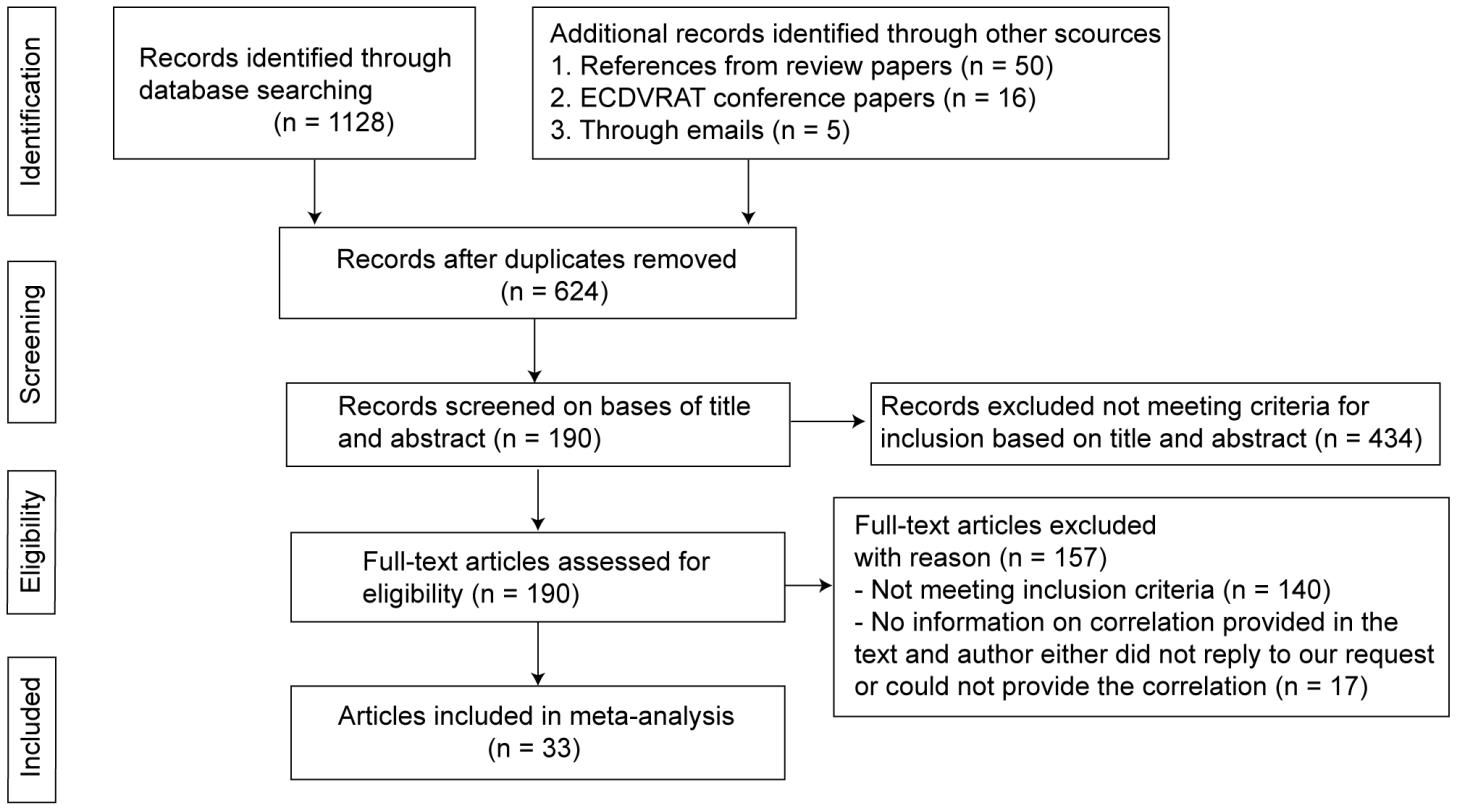
The procedure and the results of the study search and selection.

The algorithm for the selection of the studies and the results of the search are detailed in the PRISMA flow diagram [56] in Figure 1. On the basis of the standardized methodology, we included 33 articles with 52 correlations, with a total sample size of 1196 participants. All the included publications were written in English. No studies on treating PTSD [40], [57] were included because the authors of these papers could not provide the correlation between presence and anxiety experienced in the virtual environment.

The initial search identified 624 potential hits. After excluding 434 articles based on the lack of meeting the criteria after screening title and abstract, 190 articles were assessed for eligibility. 140 articles were then excluded based on the inclusion criteria as defined above. We then contacted the authors of all the remaining 50 articles with the request to provide correlations or other information, i.e., information about display technology, tracker, and participants. Finally, the authors of only 2 articles did not reply at all, the authors of 15 other articles replied, but could not provide the required correlation, the authors of 25 more articles replied with the required information, and 8 articles already had the required correlation reported in the paper. So, for all 33 articles for which the correlation information was available, most of the authors were also able to provide the additional information about the moderators. For this detailed information we refer to Table S1 and Table S2.

Thus, in total 33 articles were included in the meta-analysis using virtual reality designed for treatment of anxiety disorders. The anxiety disorders addressed in these articles (with their references between brackets) were: ***Acrophobia*** [23], [25], [42], [46], [58]–[60], ***Agoraphobia*** [48], [61], ***Claustrophobia*** [62], [63], ***Fear of animals*** [43], [64]–[70], ***Fear of flying*** [24], [41], ***Obsessive-compulsive disorder*** [26], ***Social phobia*** [27], [28], [71]–[75], and ***Exam/test anxiety*** [76]–[78]. One study investigated mixed phobias including fear of animals and Acrophobia or enclosed spaces [79]. As mentioned above, no publication on VRET for PTSD was included in the meta-analysis. In addition, we only included one study on VRET for OCD. Since OCD is not listed as an anxiety disorder in DSM-V [2], the new version published in 2013, we conducted all analyses twice, i.e., first with all 33 publications, and then with the same dataset, but without the study on OCD. The results of the analyses on moderating effects are based on the entire dataset. In cases where the analyses while excluding the study on OCD differ from the analyses of the entire dataset, the results of both analyses are reported.

**Figure 2:**
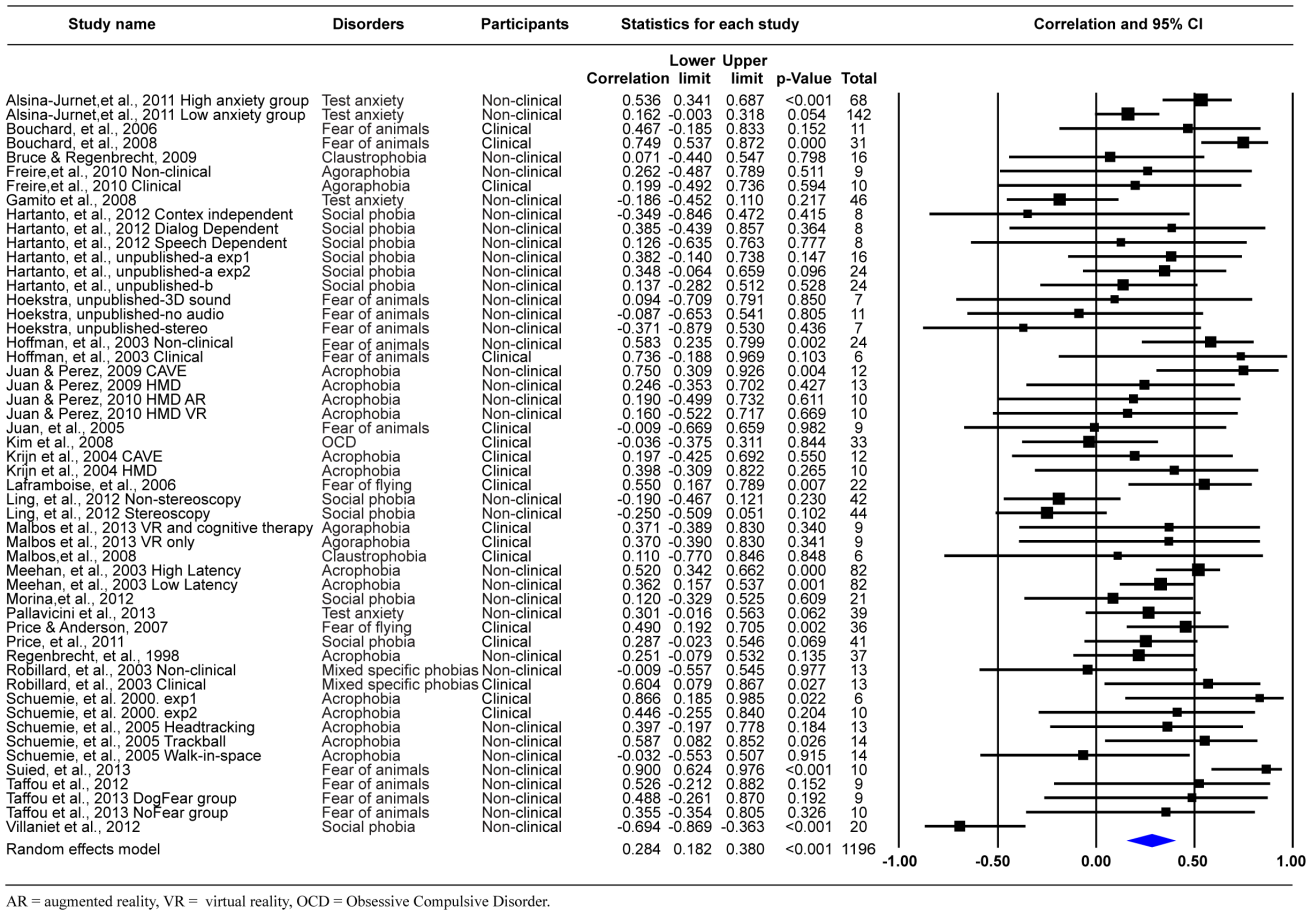
Forest plot of the correlation between presence and anxiety.

The effect size of all 33 publications including 52 correlations showed a weighted mean correlation between self-reported presence and anxiety of *r* = .28, 95% CI [0.18–0.38], *p*<.001 (see Forest Plot in Figure 2). The analysis of the dataset without the study on OCD also showed a medium effect size with a weighted mean correlation between self-reported presence and anxiety of *r* = .29, 95% CI [0.19–0.39], *p*<.001. To evaluate whether the result remained stable when smaller studies were excluded, we analyzed the summary effect after excluding studies with a sample size smaller than 15. The analysis of the dataset with 21 correlations showed a weighted mean correlation *r* = .24, 95% CI [0.09–0.38], *p* = .002, which seems to correspond to the effect estimation based on the whole dataset. Additionally, preliminary analyses indicated substantial heterogeneity in the data (fixed effects: *Q*_51_ = 126.17, *p*<.001 for the entire dataset, and *Q*_50_ = 123.04, *p*<.001 for the dataset without the study on OCD) suggesting that the data are heterogeneous, which supports the choice for a random-effects model.

In our study, the required number of studies to reduce the overall effect size to a non-significant level was 5×52+10 = 270 and 5×51+10 = 265 for the entire dataset and the dataset excluding the study on OCD, respectively according to Rosenthal [54]. The *Fail-safe N* analysis on our data showed that it would require more than 890 and 897 current or future unpublished studies on anxiety disorders with an effect size of zero for the entire dataset and the dataset without the OCD study respectively, to bring the overall effect size of the primary analysis within the non-significant range, suggesting that our meta-analysis study is robust. The shape of the funnel plots in Figure S1 did not reveal any indication of funnel plot asymmetry. This visual impression was also confirmed by Egger's test with *p* = .67, two-tailed.

### 3.1 Effect size across different measurements

We categorized the presence questionnaires used in all the 33 articles and counted the number of occurrences of each questionnaire over the 52 correlations. This yielded the following categorization (in order of number of occurrences): Igroup Presence Questionnaires [16] (N = 27), Presence Questionnaire [19], [20] (N = 10), Slater-Usoh-Steed Questionnaire [17], [18] (N = 9), one-item presence questionnaire [23], [41] (N = 3) and others [42], [43]. Note that measurements that were used only by one research group were not included in the subgroup analyses. Anxiety measurements were categorized (in order of number of occurrences) into SUD [45] (N = 36), STAI-S [44] (N = 8), and One-item question [23], [26], [46] (N = 6). The subgroup analyses showed no significant moderating effect for the type of anxiety measurement (*Q*_2_ = 1.35, *p* = .51; see Table 2), and the type of presence measurement (*Q*_4_ = 6.06, *p* = .20; see Table 2).

**Table 2:** Results of subgroup analyses using presence and anxiety as moderators.

				95% Confidence Interval	
Moderators	Number of correlations	*r*	*p*	Lower limit	Upper limit
**Presence**					
Igroup Presence Questionnaire	27	.23	.001	.09	.35
One item	3	.59	.004	.21	.82
Others	2	.18	.446	−.28	.58
Presence questionnaire	10	.32	.003	.11	.50
SUS	9	.44	<.001	.25	.60
**Anxiety**					
One item	6	.37	.005	.12	.57
STAI-S	8	.21	.065	−.01	.41
SUD	36	.34	<.001	.21	.45

### 3.2 Effect size across different anxiety disorders

Correlations between presence and anxiety were significantly different among the various disorders, varying from almost zero for social phobia and claustrophobia to a large effect for fear of animals and fear of flying (see Table 3). Subgroup analyses showed significant differences between different anxiety disorders (*Q*_6_ = 17.28, *p* = .008). For claustrophobia the summary correlation was not found to deviate significantly from zero, but this result was only based on two correlations. For fear of flying, the summary correlation was found to be significantly above zero, but still as the confidence interval shows, the exact size of the correlation is not precise. In contrast, 11 correlations were included in the calculation of the summary effect for social phobia, and still the mean correlation did not deviate significantly from zero.

**Table 3:** Results of subgroup analysis using disorder type as a moderator.

				95% Confidence Interval	
Disorders	Number of correlations	*r*	*p*	Lower limit	Upper limit
Acrophobia	14	.39	<.001	.22	.54
Agoraphobia	4	.30	.176	−.14	.64
Claustrophobia	2	.08	.792	−.48	.59
Fear of animals	12	.50	.000	.30	.66
Fear of flying	2	.52	.006	.16	.75
Social phobia	11	.001	.990	−.19	.19
Test anxiety	4	.22	.076	−.02	.44

### 3.3 Effect size across different participants' characteristics

Participants' characteristics including gender, age and clinical status (i.e., meeting the criteria for an anxiety disorder or not) were retrieved and used as moderator. The resulting model did not show a significant effect of gender on the correlation (*Q*_1_ = 1.82, *p* = .18). A similar meta-regression analysis was performed with mean age as a continuous variable, and again, age did not show a significant effect on the correlation between presence and anxiety (*Q*_1_ = 2.64, *p* = .10). Finally, we investigated the effect of the clinical status. The number of correlations in the group of studies with people meeting criteria for an anxiety disorder was 17, whereas the number of correlations in the group of studies with people not meeting criteria for an anxiety disorder was 35. The summary effect for the group of studies with non-clinical participants was *r* = .22, 95% CI [0.10–0.34], *p*<.001, while the summary effect for the group of studies using clinical participants was *r* = .42, 95% CI [0.25–0.57], *p*<.001. Hence, the correlation between presence and anxiety was higher for the clinical sample of people than for the non-clinical sample, but the effect of this moderator was only approaching significance (*Q*_1_ = 3.47, *p* = .06). The difference between non-clinical participants (*r* = .22, 95% CI [0.10–0.34], *p*<.001) and clinical participants (*r* = .47, 95% CI [0.29–0.61], *p*<.001) was, however, significant for the dataset without the study on OCD (*Q*_1_ = 5.15, *p* = .02).

### 3.4 Effect size across different technology characteristics

Subgroup analyses were conducted for moderators including display type (2 levels: HMD or CAVE), stereoscopy (2 levels: yes or no), degrees of freedom (DoF) of the tracker (2 levels: 3 or 6) and its update speed (5 levels: 60, 120, 125, 256 and 512 Hz), audio type (3 levels: no audio, stereo or 3D sound) and audio device (3 levels: headphones, PC speakers and stereo speakers). The subgroup analyses showed a significant moderating effect for the degrees of freedom of the tracker (*Q*_1_ = 4.81, *p* = .03) with a tracker with 6 DoF resulting in higher correlations than a tracker with 3 DoF (see Table 4). No significant differences were found for display type (*Q*_1_ = 1.56, *p* = .21), stereoscopy (*Q*_1_ = 0.014, *p* = .91), whether a tracker was used or not (*Q*_1_ = 2.65, *p* = .10), tracker's update frequency (*Q*_4_ = 3.58, *p* = .47), audio type (*Q*_2_ = 3.90, *p* = .14) and audio player (*Q*_2_ = 1.66, *p* = .44) (see again Table 4).

**Table 4:** Results of subgroup analysis using technology characteristics as moderators.

				95% Confidence Interval	
Moderators	Number of correlations	*r*	*p*	Lower limit	Upper limit
**Display**					
HMD	40	.26	<.001	.15	.37
CAVE four sides	5	.49	.009	.13	.73
**Stereoscopy**					
No	21	.28	.002	.11	.44
Yes	30	.29	<.001	.16	.42
**Tracker**					
No	9	.04	.831	−.29	.35
Yes	42	.32	<.001	.21	.42
**Tracker's DoF**					
3 DoF	27	.23	.001	.09	.36
6 DoF	16	.46	<.001	.30	.60
**Tracker Hz**					
60 Hz	8	.49	.006	.16	.73
120 Hz	3	.49	.074	−.05	.81
125 Hz	5	.08	.712	−.35	.49
256 Hz	7	.16	.399	−.22	.50
512 Hz	4	.38	.144	−.14	.74
**Audio**					
No	2	.13	.621	−.38	.59
Stereo	36	.23	.001	.10	.35
3D sound	7	.51	<.001	.25	.70
**Audio player**					
Headphones	29	.32	<.001	.17	.46
PC speakers	4	.16	.509	−.30	.56
Stereo speakers	9	.16	.179	−.07	.37

Meta-regression analyses were conducted for continuous moderating factors including display spatial resolution and its field of view (FOV). The results showed a non-significant relation between presence and anxiety with neither spatial resolution (*Q*_1_ = 0.90, *p* = .34) nor field of view (*Q*_1_ = 2.48, *p* = .12). However, examining the distribution of the diagonal field of view across the studies delivered three clusters: one with FOV below 71° (i.e., 40 correlations), one with FOV between 94.2° and 107° (i.e., 6 correlations), and one with the FOV equal to 270° (i.e., 5 correlations). A subgroup analysis on the correlation between presence and anxiety for the group of large field of view displays (FOV>94°) separated from the group of small field of view displays (FOV<71°) showed a significantly higher correlation (*Q*_1_ = 4.10, *p* = 04) for studies using a large field of view display *r* = .51, 95% CI [0.28–0.68], *p*<.001 compared to studies using a small field of view display *r* = .24, 95% CI [0.13–0.35], *p*<.001.

Since only tracker's DoF and display field of view significantly moderated the correlation between presence and anxiety, we conducted multiple meta-regression analyses to test whether the combination of these two variables could have a better overall prediction of the weighted mean correlation. The meta-regression analyses were done using DoF and FOV as independent variables and correlation between presence and anxiety as the dependent variable. The independent variables were entered in the order of their significance value, i.e., the tracker's DoF was entered first. As expected, the meta-regression models were all significant in both steps; *Q*_1_ = 4.51, *p* = .03 and *Q*_2_ = 7.00, *p* = .03 for step1 and step 2 individually (Table 5). The combination of DoF and FOV raised the proportion of total between-study variance from 0.12 to 0.16. The interaction effect between DoF and FOV on the correlation between presence and anxiety was not significant *Q*_1_ = 0.15, *p* = .70, which indicated that the effects of DoF and FOV were independent.

**Table 5:** Restricted maximum likelihood (REML) multiple meta-regression for field of view and tracker's DoF.

Step	Variable	*B*	*SE B*	*p*	Overall model	*R^2^*
1	DoF	0.27	0.13	.03	*Q*_1_ = 4.51, *p* = .03	0.12
2	DoF	0.15	0.15	.32	*Q*_2_ = 7.00, *p* = .03	0.16
	FOV	0.32	0.21	.12		

In summary, there seems to immerse a pattern in the effect of technology variables on the relationship between presence and anxiety. Advancements in immersive technology (i.e., higher degrees of freedom of the tracker and larger fields of view of the display) coincide with a higher correlation between presence and anxiety. A potential explanation for this observation is that advancements in immersive technology reduce the moderating effect of other factors, such as personality, clinical status and the surrounding real world. Therefore, to test whether the effect of participants' clinical status depends on display characteristics, i.e., field of view and tracker's degree of freedom, we tested the interaction effects. The results showed a significant interaction effect between participants' clinical status and display field of view (*Q*_1_ = 4.19, *p* = .04) and between participants' clinical status and tracker's degree of freedom (*Q*_1_ = 13.66, *p*<.001). The hypothesis was explored further by examining the moderating effect of participants' characteristics (i.e., clinical or non-clinical individuals) in studies that used less or more advanced immersive technology. As such, we performed a subgroup analysis separating studies with clinical or non-clinical participants for studies using trackers with 6 DoF or 3 DoF separately. The results in Table 6 show that participants' clinical status did not moderate the correlation between presence and anxiety when using advanced technology (i.e., a tracker with higher degrees of freedom; *Q*_1_ = 0.10, *p* = .76), but it did for trackers with lower degrees of freedom (*Q*_1_ = 8.38, *p* = .004). Likewise, we analyzed the effect of participants' clinical status on the relation between presence and anxiety separately for studies using a display with a large field of view and studies using a display with a small field of view. In analogy with the results of the subgroup analyses for the DoF of trackers, also these results support our hypothesis; the participants' clinical status did not moderate the correlation between presence and anxiety when using a display with a large field of view (*Q*_1_ = 0.14, *p* = .71), yet it did (*Q*_1_ = 5.69, *p* = .017) when using a display with a small field of view (see again Table 6).

**Table 6:** Results of subgroup analyses using participants' clinical status as moderators for trackers' DOF (i.e., 6 DoF and 3 DoF) and displays' field of view (i.e., large field of view and small field of view) respectively.

Moderators				95% Confidence Interval	
Clinical vs. non-clinical	Number of correlations	*r*	*p*	Lower limit	Upper limit
**6 trackers' degree of freedom**					
non-clinical group	13	.45	<.001	.33	.56
clinical group	3	.39	.095	−.07	.71
**3 trackers' degree of freedom**					
non-clinical group	14	.06	.544	−.13	.24
clinical group	12	.46	<.001	.26	.62
**Large field of view**					
non-clinical group	9	.52	<.001	.27	.71
clinical group	2	.41	.231	−.27	.82
**Small field of view**					
non-clinical group	25	.15	.025	.02	.28
clinical group	15	.42	<.001	.25	.58

### 3.5 Effect size as a function of sample size and publication year

To investigate the impact of sample size on the correlation between presence and anxiety, we performed the meta-regression analysis, using the sample size as a continuous variable and the effect size as the dependent variable. The model was not significant (*Q*_1_ = 0.07, *p* = .80), indicating that the correlation between presence and anxiety did not differ with sample size of the study.

**Figure 3:**
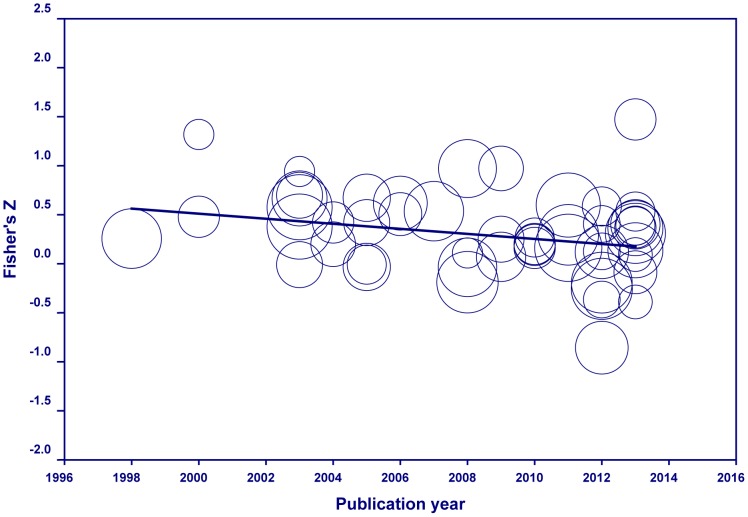
Regression of publication year on transformed value of *r*, i.e., Fisher's *Z*.

Finally, we conducted a meta-regression analysis using the publication year as a continuous variable and again the correlation between presence and anxiety as a dependent variable. This model was approaching significant (*Q*_1_ = 3.78, *p* = .051) with 9.62% of the variability in the correlation coefficients accounted for by the publication year (see Table 7). As the publication is from a later date, the correlation between presence and anxiety decreases (see Figure 3). This effect, however, may have been confounded with the type of anxiety disorder, since significantly more social phobia studies (*M* = 2012.2, *SD* = .18) compared to studies on other disorders (*M* = 2007.6, *SD* = .67, independent-sample *t*-test: *t*(50) = 3.50, *p* = .001) were reported during the more recent years, and we showed before that the correlation between presence and anxiety was almost zero for social phobia studies.

**Table 7:** Restricted maximum likelihood (REML) meta-regression for publication year moderator.

Variable	*B*	*p*
Interval	−0.026	.0051
Regression Constant	51.96	
Overall model	*Q*(1) = 3.78	
Residual	*Q*(50) = 113.28	
Total	*Q*(51) = 126.17	
*R* ^2^ = 0.0962		

## Discussion and Conclusions

The present meta-analysis, including 33 articles with 52 correlations, explored the relation between self-reported presence and anxiety during virtual reality exposure therapy for anxiety disorders. The random effects analysis showed a medium effect size for the correlation between presence and anxiety of *r* = .28 (95% CI: .18–.38). Our results indicate that, in general, self-reported presence and anxiety are moderately associated with each other during virtual reality exposure therapy for the treatment of anxiety.

One factor that influenced the effect size was disorder type. For disorders like acrophobia (*r* = .39), fear of animals (*r* = .50), and fear of flying (*r* = .52), correlations between presence and anxiety were all above zero, with fear of animals and fear of flying showing a large effect size. However, for social phobia no correlation between self-reported presence and anxiety was found. As several trials have demonstrated the potential of VRET for social anxiety disorder [27], [28], [75], one might conclude that the current subjective presence measures do not capture the essential sense of presence that is responsible for activating fear related to social anxiety in individuals. Social anxiety centers on the perception of negative human evaluation, and thus individuals' perception of the appearance of both verbal and non-verbal behavior of virtual characters [80] seems a relevant cue for activating the fear structure. However, most self-reported spatial presence measurements used in the studies we evaluated here focus on individuals' feelings of being present in a certain location or place. This might, therefore, explain correlations with medium and large size effect found for anxiety disorders such as acrophobia and fear of flying where the activation of the fear structure may be cues of certain locations such as a virtual balcony or the inside of an virtual airplane. Recently, Slater [81] also argued that presence at least has two independent components: place illusion and plausibility. Similar to physical presence, place illusion refers to the feeling of being in the virtual environment. Plausibility is the illusion that what is happening in the virtual world is really happening in spite of the knowledge that it is mediated technology. Both place illusion and plausibility contribute to realistic responses in the virtual environment. As suggested above, place illusion is well covered by most presence questionnaires, but future research should investigate the extent to which presence questionnaires also measure plausibility, i.e., the extent to which one takes what is happening in the virtual environment as real, since the latter might be more relevant than spatial presence for VRET for social phobia. There might be a second reason for the low correlation between presence and anxiety in some studies. Bouchard et al. [82] argued that there might exist an unknown trigger point where the level of presence is sufficient to lead to a strong sense of anxiety, and increasing presence only contributes moderately to the increase in anxiety, indicating that the relationship between presence and anxiety might not be linear. Our current meta-analysis study, however, was based on correlations between presence and anxiety, where the shape of the relationship between presence and anxiety could not be modeled.

Another factor that influenced the effect size of the correlation between presence and anxiety was the participants' clinical status (i.e., meeting criteria for an anxiety disorder or not). The correlation reported for clinical samples appeared larger than for non-clinical samples. This finding seems plausible as real life anxiety-related cues can much easier activate anxiety reactions in individuals with an anxiety disorder than in individuals without an anxiety disorder [11]. Similarly, virtual environments that resemble feared situations in real life can have a stronger impact on individuals with an anxiety disorder than on individuals without an anxiety disorder. Nonetheless, for both clinical and non-clinical individuals, the reported presence can be seen as the degree in which real life cues have been imitated. For non-clinical individuals the presence of these cues might lead to the activation of a fear structure that does not include an intense fear response. The finding that sense of presence is significantly correlated with anxiety levels not only in clinical samples but also in non-clinical samples can prove relevant for non-treatment research on anxiety. As a consequence, basic research can effectively apply a virtual setting to elicit fear and thereby examine potential features associated with anxiety. It should be noted however, that anxiety reported in a virtual environment might not only be related to the presence of anxiety provoking cues, since other factors such as simulation sickness may also elicit some level of anxiety [83]–[85].

The meta-analysis also found moderating effects for some technology characteristics such as the degrees of freedom of the tracker and the display's field of view. Higher levels of immersive technology coincide with higher correlations between presence and anxiety. These findings put forward the hypothesis that applying more advanced immersive technology leaves less room for other factors to moderate the correlation between presence and anxiety. Such a factor could be the individuals' clinical status (i.e., clinical vs. non-clinical participants). Examining the decrease in the moderating effect of the individuals' clinical status from studies with less (i.e., trackers with 3 degree of freedom and displays with smaller field of view) to studies with more (i.e., trackers with 6 degree of freedom and displays with larger field of view) advanced immersive technology, confirmed this hypothesis. The moderating effect was found in the studies with less immersive technology, but not in studies with more immersive technology. It seems that advanced immersive technology dominates the relationship and reduces the ‘noise’ from other factors such as individuals' characteristics, personality or previous experiences which may affect experienced presence and anxiety in virtual environments. This finding is also in line with what Ling et al. [86] suggested, namely that the role of participants' absorption may be larger in less immersive virtual environments. This hypothesis would encourage the use of more advanced immersive technology for VRET to reduce the effect of less controllable factors on presence, such as individuals' visual acuity and immersive tendency [86]. Additionally, as the lack of emotional response has been reported as a potential cause for dropouts in VRET [25], [87], and the use of advanced technology increases the correlation between presence and anxiety, we would expect lower amount of dropouts in VRET by using advanced technology.

This meta-analysis has some limitations. First, the measurements of presence and anxiety used in this study were self-reported which is prone to well-known demand characteristics. Participants may guess what the researchers examine and which outcome they expect, and then answer accordingly or contradictory to these expectations [88]. Second, a very limited number of studies for obsessive-compulsive disorder, claustrophobia and fear of flying were included in the meta-analysis. Therefore, caution should be taken with generalizing the findings to underrepresented disorders in this meta-analysis. Yet, for acrophobia and fear of animals we can draw the conclusion that the effect size of correlation between presence and anxiety ranges from medium to large.

To conclude, the main finding of the meta-analysis is that self-reported presence has a medium size association with self-reported anxiety in VRET, justifying research into presence improvement.

## Supporting Information

Figure S1 Funnel plot for publication bias test.
(TIF)

Table S1 Characteristics of studies included in the meta-analysis: measurements, disorders and participants' characteristics.
(PDF)

Table S2 Characteristics of studies included in the meta-analysis: technology characteristics.
(PDF)

Checklist S1 PRISMA checklist.
(DOC)
