# Supplementary figures and images for: A Meta-Analysis on the Relationship between Self-Reported Presence and Anxiety in Virtual Reality Exposure Therapy for Anxiety Disorders
Source: PLoS One. 2014 May 6;9(5):e96144. doi: 10.1371/journal.pone.0096144 (PMC4011738; doi:10.1371/journal.pone.0096144)

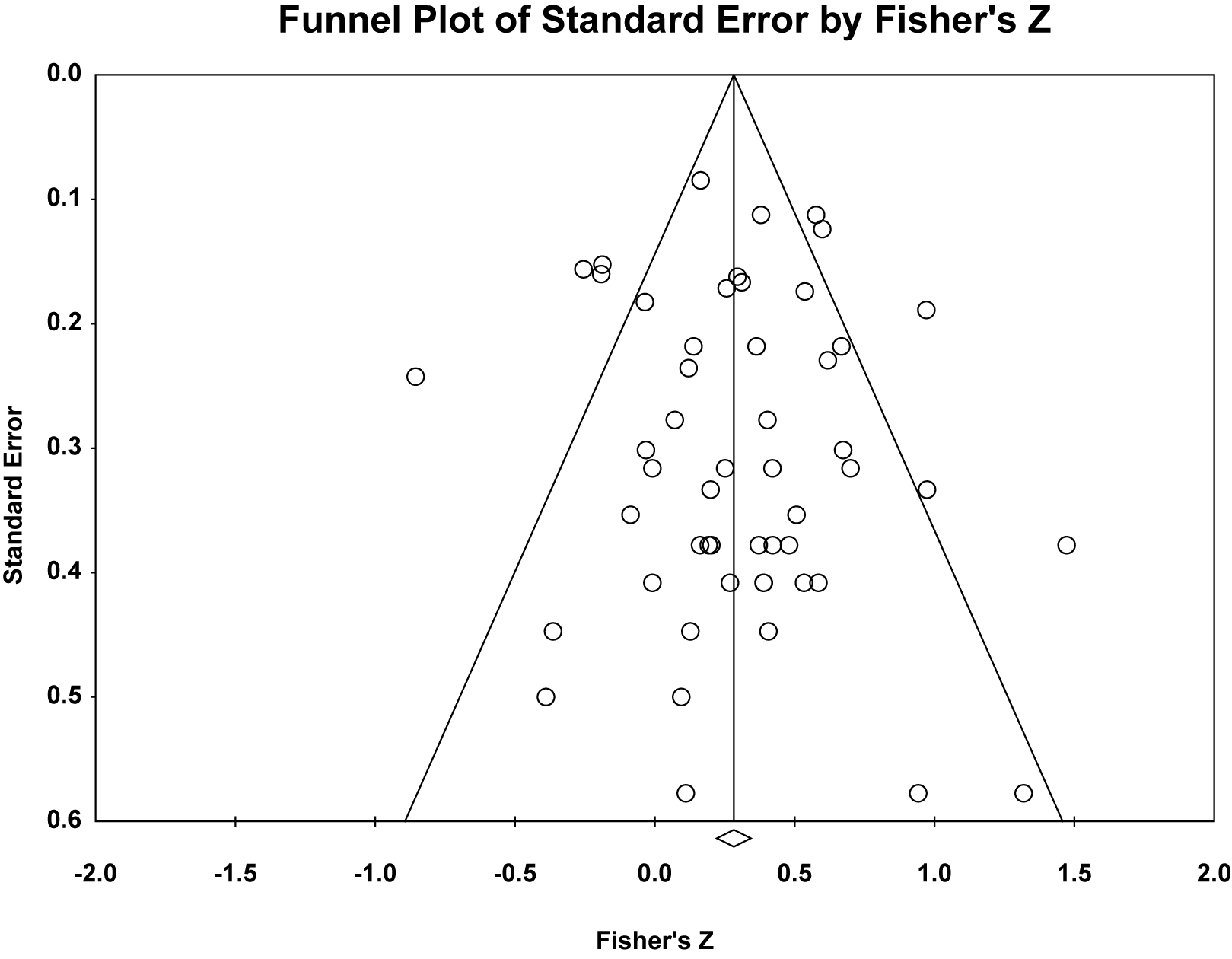

Supplement: Figure S1 — Funnel plot for publication bias test. (TIF) [file pone.0096144.s001.tif]
