# Supplementary material for: A Meta-Analysis on the Relationship between Self-Reported Presence and Anxiety in Virtual Reality Exposure Therapy for Anxiety Disorders
Source: PLoS One. 2014 May 6;9(5):e96144. doi: 10.1371/journal.pone.0096144 (PMC4011738; doi:10.1371/journal.pone.0096144)
Supplement: Table S1 — Characteristics of studies included in the meta-analysis: measurements, disorders and participants' characteristics. (PDF) [file pone.0096144.s002.pdf]

# A meta-analysis on the relationship between self-reported presence and anxiety in virtual reality exposure therapy for anxiety disorders

Yun Ling, Harold T. Nefs, Nexhmedin Morina, Ingrid Heynderickx, Willem-Paul Brinkman

## Table S1: Characteristics of studies included in this meta-analysis: measurements, disorders and participants' characteristics

| Article No. | Study name | Correlation | Sample size | Presence questionnaire | Anxiety | Disorders | Participants | Male/sum | age mean (years) |
|---|---|---|---|---|---|---|---|---|---|
| 1.00 | Alsina-Jurnet,et al., 2011 High test anxiety group | 0.54 | 68.00 | Igroup Presence Questionnaire (IPQ) | STAI-S | Test anxiety | Non-Phobic | 0.12 | 22.35 |
| 1.00 | Alsina-Jurnet,et al., 2011 Low test anxiety group | 0.16 | 142.00 | Igroup Presence Questionnaire (IPQ) | STAI-S | Test anxiety | Non-Phobic | 0.20 | 23.58 |
| 2.00 | Bouchard, et al., 2006 | 0.47 | 11.00 | Presence questionnaire | SUD | Fear of animals | Phobic | 0.09 | 30.73 |
| 3.00 | Bouchard, et al., 2008 | 0.75 | 31.00 | Presence questionnaire | STAI-S | Fear of animals | Phobic | 0.16 | 44.97 |
| 4.00 | Bruce & Regenbrecht, 2009 HMD and monitor | 0.07 | 16.00 | Igroup Presence Questionnaire (IPQ) | SUD | Claustrophobia | Non-Phobic | 0.67 | 28.40 |
| 5.00 | Freire,et al., 2010 Non-clinical | 0.26 | 9.00 | Igroup Presence Questionnaire (IPQ) | SUD | Agoraphobia | Non-Phobic | 0.33 | 37.70 |
| 5.00 | Freire,et al., 2010 Clinical | 0.20 | 10.00 | Igroup Presence Questionnaire (IPQ) | SUD | Agoraphobia | Phobic | 0.40 | 39.20 |
| 6.00 | Gamito et al., 2008 | -0.19 | 46.00 | Presence questionnaire | STAI-S | Test anxiety | Non-Phobic | 0.26 | 22.36 |
| 7.00 | Hartanto, et al., 2012 Contex independent | -0.35 | 8.00 | Igroup Presence Questionnaire (IPQ) | SUD | Social phobia | Non-Phobic | 0.50 | 29.00 |
| 7.00 | Hartanto, et al., 2012 Dialog Dependent | 0.39 | 8.00 | Igroup Presence Questionnaire (IPQ) | SUD | Social phobia | Non-Phobic | 0.50 | 30.30 |
| 7.00 | Hartanto, et al., 2012 Speech Dependent | 0.13 | 8.00 | Igroup Presence Questionnaire (IPQ) | SUD | Social phobia | Non-Phobic | 0.63 | 31.60 |
| 8.00 | Hartanto, et al., unpublished-a exp1 | 0.38 | 16.00 | Igroup Presence Questionnaire (IPQ) | SUD | Social phobia | Non-Phobic | 0.69 | 22.44 |
| 8.00 | Hartanto, et al., unpublished-a exp2 | 0.35 | 24.00 | Igroup Presence Questionnaire (IPQ) | SUD | Social phobia | Non-Phobic | 0.46 | 29.37 |
| 9.00 | Hartanto, et al., unpublished-b | 0.14 | 24.00 | Igroup Presence Questionnaire (IPQ) | SUD | Social phobia | Non-Phobic | 0.38 | Blank |
| 10.00 | Hoekstra, unpublished-3D sound | 0.09 | 7.00 | Igroup Presence Questionnaire (IPQ) | SUD | Fear of animals | Non-Phobic | 0.43 | 28.14 |
| 10.00 | Hoekstra, unpublished-no audio | -0.09 | 11.00 | Igroup Presence Questionnaire (IPQ) | SUD | Fear of animals | Non-Phobic | 0.73 | 26.18 |
| 10.00 | Hoekstra, unpublished-stereo | -0.37 | 7.00 | Igroup Presence Questionnaire (IPQ) | SUD | Fear of animals | Non-Phobic | 0.71 | 32.29 |
| 11.00 | Hoffman, et al., 2003 Non-clinical | 0.58 | 24.00 | SUS | SUD | Fear of animals | Non-Phobic | 0.21 | 18.79 |
| 11.00 | Hoffman, et al., 2003 Clinical | 0.74 | 6.00 | SUS | SUD | Fear of animals | Phobic | 0.00 | 18.33 |
| 12.00 | Juan & Perez, 2009 CAVE | 0.75 | 12.00 | SUS | SUD | Acrophobia | Non-Phobic | 0.83 | 23.33 |
| 12.00 | Juan & Perez, 2009 HMD | 0.25 | 13.00 | SUS | SUD | Acrophobia | Non-Phobic | 0.77 | 25.54 |
| 13.00 | Juan & Perez, 2010 HMD AR | 0.19 | 10.00 | SUS | SUD | Acrophobia | Non-Phobic | 0.70 | 28.20 |
| 13.00 | Juan & Perez, 2010 HMD VR | 0.16 | 10.00 | SUS | SUD | Acrophobia | Non-Phobic | 0.90 | 27.80 |
| 14.00 | Juan, et al., 2005 | -0.01 | 9.00 | Others | SUD | Fear of animals | Phobic | 0.11 | 27.56 |
| 15.00 | Kim et al., 2008 | -0.04 | 33.00 | Presence questionnaire | One item | Obsessive-compulsive disorder (OCD) | Phobic | 0.79 | 29.90 |
| 16.00 | Krijn et al., 2004 CAVE | 0.20 | 12.00 | Igroup Presence Questionnaire (IPQ) | STAI-S | Acrophobia | Phobic | 0.25 | 52.08 |
| 16.00 | Krijn et al., 2004 HMD | 0.40 | 10.00 | Igroup Presence Questionnaire (IPQ) | STAI-S | Acrophobia | Phobic | 0.80 | 50.50 |
| 17.00 | Laframboise, et al., 2006 | 0.55 | 22.00 | One item | SUD | Fear of flying | Phobic | 0.32 | 44.74 |
| 18.00 | Ling, et al., 2012 2D | -0.19 | 42.00 | Igroup Presence Questionnaire (IPQ) | MPRCS | Social phobia | Non-Phobic | 0.55 | 26.83 |
| 18.00 | Ling, et al., 2012 3D | -0.25 | 44.00 | Igroup Presence Questionnaire (IPQ) | MPRCS | Social phobia | Non-Phobic | 0.64 | 29.20 |
| 19.00 | Malbos et al., 2013 VR and cognitive therapy | 0.37 | 9.00 | Presence questionnaire | SUD | Agoraphobia | Phobic | 0.33 | 49.22 |
| 19.00 | Malbos et al., 2013 VR only | 0.37 | 9.00 | Presence questionnaire | SUD | Agoraphobia | Phobic | 0.44 | 39.00 |
| 20.00 | Malbos,et al., 2008 | 0.11 | 6.00 | Presence questionnaire | SUD | Claustrophobia | Phobic | 0.33 | 44.33 |
| 21.00 | Meehan, et al., 2003 High Latency | 0.52 | 82.00 | SUS | One item | Acrophobia | Non-Phobic | 0.82 | 35.20 |
| 21.00 | Meehan, et al., 2003 Low Latency | 0.36 | 82.00 | SUS | One item | Acrophobia | Non-Phobic | 0.86 | 35.90 |
| 22.00 | Morina,et al., 2012 | 0.12 | 21.00 | Igroup Presence Questionnaire (IPQ) | SUD | Social phobia | Non-Phobic | 0.33 | 22.67 |
| 23.00 | Pallavicini et al., 2013 | 0.30 | 39.00 | SUS | One item | Test anxiety | Non-Phobic | 0.36 | 21.10 |
| 24.00 | Price & Anderson, 2007 | 0.49 | 36.00 | Presence questionnaire | SUD | Fear of flying | Phobic | 0.15 | 39.00 |
| 25.00 | Price, et al., 2011 | 0.29 | 41.00 | Igroup Presence Questionnaire (IPQ) | SUD | Social phobia | Phobic | 0.40 | 37.82 |
| 26.00 | Regenbrecht, et al., 1998 | 0.25 | 37.00 | Others | STAI-S | Acrophobia | Non-Phobic | 0.62 | 27.00 |
| 27.00 | Robillard, et al., 2003 Non-clinical | -0.01 | 13.00 | Presence questionnaire | SUD | Mixed specific phobias | Non-Phobic | 0.31 | 33.90 |
| 27.00 | Robillard, et al., 2003 Clinical | 0.60 | 13.00 | Presence questionnaire | SUD | Mixed specific phobias | Phobic | 0.31 | 33.70 |

| | | | | | | | | | | |
|---|---|---|---|---|---|---|---|---|---|---|
| 28.00 | Schuemie, et al. 2000. exp1 | 0.87 | 6.00 | One item | One item | Acrophobia | Phobic | 0.50 | Blank |
| 28.00 | Schuemie, et al. 2000. exp2 | 0.45 | 10.00 | One item | One item | Acrophobia | Phobic | 0.30 | Blank |
| 29.00 | Schuemie, et al., 2005 Headtracking | 0.40 | 13.00 | Igroup Presence Questionnaire (IPQ) | SUD | Acrophobia | Non-Phobic | 0.38 | 30.62 |
| 29.00 | Schuemie, et al., 2005 Trackball | 0.59 | 14.00 | Igroup Presence Questionnaire (IPQ) | SUD | Acrophobia | Non-Phobic | 0.50 | 26.50 |
| 29.00 | Schuemie, et al., 2005 Walk-in-space | -0.03 | 14.00 | Igroup Presence Questionnaire (IPQ) | SUD | Acrophobia | Non-Phobic | 0.50 | 34.14 |
| 30.00 | Suied, et al., 2013 | 0.90 | 10.00 | Igroup Presence Questionnaire (IPQ) | SUD | Fear of animals | Non-Phobic | 0.40 | 33.00 |
| 31.00 | Taffou et al., 2012 | 0.53 | 9.00 | Igroup Presence Questionnaire (IPQ) | SUD | Fear of animals | Non-Phobic | 0.44 | 36.60 |
| 32.00 | Taffou et al., 2013 DogFear group | 0.49 | 9.00 | Igroup Presence Questionnaire (IPQ) | SUD | Fear of animals | Non-Phobic | 0.44 | 36.60 |
| 32.00 | Taffou et al., 2013 NoFear group | 0.36 | 10.00 | Igroup Presence Questionnaire (IPQ) | SUD | Fear of animals | Non-Phobic | 0.60 | 32.50 |
| 33.00 | Villaniet et al., 2012 | -0.69 | 20.00 | ITC-SOPI | STAI-S | Social phobia | Non-Phobic | 0.50 | 24.00 |
