# Supplementary material for: A Meta-Analysis on the Relationship between Self-Reported Presence and Anxiety in Virtual Reality Exposure Therapy for Anxiety Disorders
Source: PLoS One. 2014 May 6;9(5):e96144. doi: 10.1371/journal.pone.0096144 (PMC4011738; doi:10.1371/journal.pone.0096144)
Supplement: Table S2 — Characteristics of studies included in the meta-analysis: technology characteristics. (PDF) [file pone.0096144.s003.pdf]

# A meta-analysis on the relationship between self-reported presence and anxiety in virtual reality exposure therapy for anxiety disorders

Yun Ling, Harold T. Nefs, Nexhmedin Morina, Ingrid Heynderickx, Willem-Paul Brinkman

## Table S2: Characteristics of studies included in this meta-analysis: technology characteristics

| Article No. | Study name | Display | Stereoscopy | FOV diagonal | Resolution | Tracker | Tracker DOF | Tracker update Hz | Audio | Audio device |
|---|---|---|---|---|---|---|---|---|---|---|
| 1.00 | Alsina-Jurnet,et al., 2011 High test anxiety group | HMD | No | 31.00 | 800*600 | yes | 3 DoF | Blank | Blank | Stereospeakers |
| 1.00 | Alsina-Jurnet,et al., 2011 Low test anxiety group | HMD | No | 31.00 | 800*600 | yes | 3 DoF | Blank | Blank | Stereospeakers |
| 2.00 | Bouchard, et al., 2006 | HMD | No | 26.00 | 640*480 | yes | 3 DoF | 256 Hz | Stereo | Headphones |
| 3.00 | Bouchard, et al., 2008 | HMD | No | 26.00 | 640*480 | yes | 3 DoF | 256 Hz | Stereo | Headphones |
| 4.00 | Bruce & Regenbrecht, 2009 HMD and monitor | Blank | Blank | Blank | Blank | Blank | Blank | Blank | Blank | Blank |
| 5.00 | Freire,et al., 2010 Non-clinical | Computer screen | No | 28.50 | 800*600 | no | Blank | Blank | Stereo | Headphones |
| 5.00 | Freire,et al., 2010 Clinical | Computer screen | No | 28.50 | 800*600 | no | Blank | Blank | Stereo | Headphones |
| 6.00 | Gamito et al., 2008 | HMD | Yes | 26.00 | 800*600 | yes | 3 DoF | 256 Hz | Stereo | Headphones |
| 7.00 | Hartanto, et al., 2012 Contex independent | Projector | No | 94.15 | 1280*1024 | no | Blank | Blank | Stereo | Headphones |
| 7.00 | Hartanto, et al., 2012 Dialog Dependent | Projector | No | 94.15 | 1280*1024 | no | Blank | Blank | Stereo | Headphones |
| 7.00 | Hartanto, et al., 2012 Speech Dependent | Projector | No | 94.15 | 1280*1024 | no | Blank | Blank | Stereo | Headphones |
| 8.00 | Hartanto, et al., unpublished-a exp1 | HMD | Yes | 40.00 | 800*600 | yes | 3 DoF | 125 Hz | Stereo | Stereospeakers |
| 8.00 | Hartanto, et al., unpublished-a exp2 | HMD | Yes | 51.60 | 1280*720 | yes | 3 DoF | Blank | Stereo | Stereospeakers |
| 9.00 | Hartanto, et al., unpublished-b | HMD | Yes | 51.60 | 1280*720 | yes | 3 DoF | 500 Hz | Stereo | Headphones |
| 10.00 | Hoekstra, unpublished-3D sound | HMD | Yes | 51.60 | 1280*720 | no | Blank | Blank | 3D spatialized sound | Headphones |
| 10.00 | Hoekstra, unpublished-no audio | HMD | Yes | 51.60 | 1280*720 | no | Blank | Blank | No | Blank |
| 10.00 | Hoekstra, unpublished-stereo | HMD | Yes | 51.60 | 1280*720 | no | Blank | Blank | Stereo | Headphones |
| 11.00 | Hoffman, et al., 2003 Non-clinical | HMD | Yes | 107.20 | 345*259 | yes | 6 DoF | 120 Hz | Stereo | Headphones |
| 11.00 | Hoffman, et al., 2003 Clinical | HMD | Yes | 107.20 | 345*259 | yes | 6 DoF | 120 Hz | Stereo | Headphones |
| 12.00 | Juan & Perez, 2009 CAVE | CAVE four sides | Yes | 270.00 | 4*1280*1024 | yes | 6 DoF | 512 Hz | Stereo | Laptop speakers |
| 12.00 | Juan & Perez, 2009 HMD | HMD | Yes | 40.00 | 800*600 | yes | 3 DoF | 512 Hz | Stereo | PC speakers |
| 13.00 | Juan & Perez, 2010 HMD AR | HMD | Yes | 40.00 | 800*600 | yes | 3 DoF | 512 Hz | Stereo | PC speakers |
| 13.00 | Juan & Perez, 2010 HMD VR | HMD | Yes | 40.00 | 800*600 | yes | 3 DoF | 512 Hz | Stereo | PC speakers |
| 14.00 | Juan, et al., 2005 | HMD | No | 40.00 | 800*600 | no | Blank | Blank | Stereo | PC speakers |
| 15.00 | Kim et al., 2008 | HMD | No | 40.00 | 800*600 | yes | 3 DoF | 60 Hz | Stereo | Stereospeakers |
| 16.00 | Krijn et al., 2004 CAVE | CAVE four sides | Yes | 270.00 | Blank | yes | 6 DoF | 144 Hz | Blank | Blank |
| 16.00 | Krijn et al., 2004 HMD | HMD | Yes | 70.50 | 640*480 | yes | 6 DoF | 144 Hz | Blank | Blank |
| 17.00 | Laframboise, et al., 2006 | HMD | No | 24.00 | 640*480 | yes | 3 DoF | Blank | Stereo | Headphones |
| 18.00 | Ling, et al., 2012 2D | HMD | Yes | 40.00 | 800*600 | yes | 3 DoF | 125 Hz | Stereo | Stereospeakers |
| 18.00 | Ling, et al., 2012 3D | HMD | Yes | 40.00 | 800*600 | yes | 3 DoF | 125 Hz | Stereo | Stereospeakers |
| 19.00 | Malbos et al., 2013 VR and cognitive therapy | HMD | No | 42.00 | 800*600 | yes | 3 DoF | 125 Hz | Stereo | Headphones |
| 19.00 | Malbos et al., 2013 VR only | HMD | No | 42.00 | 800*600 | yes | 3 DoF | 125 Hz | Stereo | Headphones |
| 20.00 | Malbos,et al., 2008 | HMD | No | 35.00 | 800*600 | yes | 3 DoF | 256 Hz | Stereo | Headphones |
| 21.00 | Meehan, et al., 2003 High Latency | HMD | Yes | 60.00 | 640*480 | yes | 6 DoF | 160 Hz | 3D spatialized sound | A virtual wall-mouted speaker |
| 21.00 | Meehan, et al., 2003 Low Latency | HMD | Yes | 60.00 | 640*480 | yes | 6 DoF | 160 Hz | 3D spatialized sound | A virtual wall-mouted speaker |
| 22.00 | Morina,et al., 2012 | HMD | Yes | 60.00 | 1280*1024 | yes | 3 DoF | 180 Hz | Stereo | Stereospeakers |
| 23.00 | Pallavicini et al., 2013 | HMD | Yes | 31.00 | 640*480 | yes | 6 DoF | 60 Hz | Stereo | Stereospeakers |
| 24.00 | Price & Anderson, 2007 | HMD | Yes | 45.00 | 263*230*3 | yes | 3 DoF | Blank | Stereo | Headphones |
| 25.00 | Price, et al., 2011 | HMD | Yes | 45.00 | 263*230*3 | yes | 3 DoF | Blank | Stereo | Headphones |
| 26.00 | Regenbrecht, et al., 1998 | HMD | No | 60.00 | 742*230 | yes | 6 DoF | 120 Hz | No | Blank |
| 27.00 | Robillard, et al., 2003 Non-clinical | HMD | No | 26.00 | 640*480 | yes | 3 DoF | 256 Hz | Stereo | Headphones |
| 27.00 | Robillard, et al., 2003 Clinical | HMD | No | 26.00 | 640*480 | yes | 3 DoF | 256 Hz | Stereo | Headphones |

| | | | | | | | | | | |
|---|---|---|---|---|---|---|---|---|---|---|
| 28.00 | Schuemie, et al. 2000. exp1 | HMD | No | 30.00 | 789*230 | yes | 3 DoF | 60 Hz | Blank | Blank |
| 28.00 | Schuemie, et al. 2000. exp2 | HMD | No | 30.00 | 789*230 | yes | 3 DoF | 60 Hz | Blank | Blank |
| 29.00 | Schuemie, et al., 2005 Headtracking | HMD | Yes | 70.50 | 640*480 | yes | 6 DoF | 144 Hz | Stereo | Headphones |
| 29.00 | Schuemie, et al., 2005 Trackball | HMD | Yes | 70.50 | 640*480 | yes | 6 DoF | 144 Hz | Stereo | Headphones |
| 29.00 | Schuemie, et al., 2005 Walk-in-space | HMD | Yes | 70.50 | 640*480 | yes | 6 DoF | 144 Hz | Stereo | Headphones |
| 30.00 | Suied, et al., 2013 | 3D passive screen | Yes | 102.00 | 1280*960 | yes | 6 DoF | 60 Hz | 3D spatialized sound | Headphones |
| 31.00 | Taffou et al., 2012 | CAVE four sides | Yes | 270.00 | 4*1600*1200 | yes | 6 DoF | 60 Hz | 3D spatialized sound | Headphones |
| 32.00 | Taffou et al., 2013 DogFear group | CAVE four sides | Yes | 270.00 | 4*1600*1200 | yes | 6 DoF | 60 Hz | 3D spatialized sound | Headphones |
| 32.00 | Taffou et al., 2013 NoFear group | CAVE four sides | Yes | 270.00 | 4*1600*1200 | yes | 6 DoF | 60 Hz | 3D spatialized sound | Headphones |
| 33.00 | Villaniet et al., 2012 | HMD | No | 35.00 | 832*624 | yes | 3 DoF | 256 Hz | Stereo | Headphones |
